# Compliance With US Federal Regulations on Waterpipe Tobacco Warnings on Packaging

**DOI:** 10.1001/jamanetworkopen.2023.54467

**Published:** 2024-02-02

**Authors:** Jennifer Cornacchione Ross, Alexandra R. Zizzi, Cynthia K. Suerken, Caroline Kimes, Eric Soule, Erin L. Sutfin

**Affiliations:** 1 Department of Social Sciences and Health Policy, Wake Forest University School of Medicine, Winston Salem, North Carolina; 2 Now with Department of Health Law, Policy and Management, Boston University School of Public Health, Boston, Massachusetts; 3 Department of Biostatistics and Data Science, Wake Forest University School of Medicine, Winston Salem, North Carolina; 4 Department of Health Education and Promotion, East Carolina University, Greenville, North Carolina

## Abstract

This cohort study reports the rate and degree of compliance with the Food and Drug Administration–mandated warning labels among waterpipe tobacco brands and products.

## Introduction

Waterpipe tobacco (WT) smoking is a public health concern, especially among young adults, who have the highest use rates.^1,2,3^ Warning labels communicate tobacco-use harms to consumers.^4^ In 2018, a nicotine warning on all WT packaging was mandated by the US Food and Drug Administration (FDA).^5^ This study assessed compliance with FDA warning requirements among WT manufacturers in the US.

## Methods

In 2020, we conducted a comprehensive online search using multiple search engines to identify all WT brands available for purchase online. We identified 66 brands, including 89 distinct collections or product lines (ie, different packaging under the same brand [eg, Starbuzz Bold, Starbuzz Vintage]). We used wave 4 (2016-2017, most recent dataset available) of the Population Assessment of Tobacco and Health^6^ study to identify 10 brands with the highest prevalence (which included 23 product lines), and we randomly selected 24 additional product lines to include. We purchased 4 randomly selected flavors from each product line, pending availability, resulting in 181 WT packages from 46 product lines representing 33 brands. The Wake Forest University School of Medicine Institutional Review Board deemed this cohort study exempt from review and informed consent because it was non–human participant research. We followed the STROBE reporting guideline.

All WT packages sold in the US are required by the FDA to display “WARNING: This product contains nicotine. Nicotine is an addictive chemical” on 2 principal panels (front or top/back). To comply, the warning must meet 7 formatting requirements: occupy at least 30% or approximately one-third of the panel, include text that takes up the largest proportion of warning area, stand out from the rest of the packaging, have centered text, have text printed in black on white background or white on black background, use correct capitalization and punctuation, and place text in the same direction as the brand.

We coded each package for FDA requirement compliance (yes or no) for up to 2 nicotine warnings. Two coders independently coded each package; a third coder (C.K.) resolved any discrepancies. We computed frequencies and percentages for all coded data. Data analysis was performed between November 2022 and December 2023 using SAS 9.4 (SAS Institute Inc).

## Results

Ninety-seven of 181 WT packages (53.6%) had the FDA-required nicotine warning statement. Among 97 packages, 68 (70.1%) had 2 warnings (37.5% of the full sample). Sixty-two packages (63.9%) had the warning on primary or front display (34.3% of full sample). Of these 62 packages, 56 (90.3%; 30.9% of full sample) placed a second warning on secondary display. Among packages without the warning on primary display (35 of 97 [36.1%]), the warnings were located on secondary (31 [32.0%]) or tertiary (4 [4.1%]) displays.

There were 165 total FDA-required nicotine warnings displayed on 97 packages. Most packages complied with all formatting elements (Table 1). The element with the least compliance was the 30%-of-panel-size requirement (120 packages; 72.7% compliant). Among 33 brands in the sample, 8 (24.2%) had 2 warnings on all of their packages, 23 (69.7%) had at least 1 warning on at least 1 package, and 10 (30.3%) had no packages with any warning (Table 2). Three brands (9.1%) had 100% compliance with all 7 formatting elements and had the required 2 warnings on their packages and in the correct location. There were some differences within brands with multiple product lines (Table 2).

**Table 1.** Characteristics of and Compliance With Nicotine Warning Formatting^a^

Characteristic	Compliance, No. (%)
Warning area at least 30% of panel	120 (72.7)
Warning text takes up largest proportion of warning area	147 (89.1)
Warning stands out from other text on package	132 (80.0)
Warning text in black font on white background or white font on black background	147 (89.1)
Warning text capitalized and punctuated as written	144 (87.3)
Warning text centered	160 (97.0)
Warning text same direction as brand text	165 (100)

^a^ 165 warnings across 97 packages that had at least 1 US Food and Drug Administration–mandated nicotine warning.

**Table 2.** Nicotine Warning Compliance by 33 Brands

Brand	No. of packages (n = 181)	No. of packages with nicotine warnings (n = 97)	No. of packages with 2 nicotine warnings per package (%)^a^	Total No. of nicotine warnings	All warnings in correct location, yes/no^b^	Formatting elements, No. (%)								Cumulative Compliance Score^e^
						30% of panel^c^	Largest proportion of label	Stand out	Black/white font/background	Correct punctuation and capitalization	Centered	Same direction as brand	Warnings with all formatting compliant^d^	
Al Amir	4	2	0	2	Yes	2 (100)	2 (100)	2 (100)	2 (100)	2 (100)	2 (100)	2 (100)	2 (100)	7
Al Fakhamah	4	0	NA	NA	NA	NA	NA	NA	NA	NA	NA	NA	NA	NA
Al Fakher [total]	12	12	12 (100)	24	Yes	24 (100)	24 (100)	24 (100)	24 (100)	24 (100)	24 (100)	24 (100)	24 (100)	8^f^
Crafted Batch^g^	4	4	4 (100)	8	Yes	8 (100)	8 (100)	8 (100)	8 (100)	8 (100)	8 (100)	8 (100)	8 (100)	8^f^
Golden Range^g^	4	4	4 (100)	8	Yes	8 (100)	8 (100)	8 (100)	8 (100)	8 (100)	8 (100)	8 (100)	8 (100)	8^f^
Standard Range^g^	4	4	4 (100)	8	Yes	8 (100)	8 (100)	8 (100)	8 (100)	8 (100)	8 (100)	8 (100)	8 (100)	8^f^
Alchemist Blend [total]	8	4	0	4	No	0	0	0	0	4 (100)	4 (100)	4 (100)	0	3
KFC^g^	4	4	0	4	No	0	0	0	0	4 (100)	4 (100)	4 (100)	0	3
Original, Stout^g^	4	0	NA	NA	NA	NA	NA	NA	NA	NA	NA	NA	NA	NA
Amy Gold	4	4	0	4	No	4 (100)	4 (100)	4 (100)	4 (100)	0	4 (100)	4 (100)	0	6
Baja	4	4	3 (75.0)	7	No	7 (100)	7 (100)	0	2 (28.6)	7 (100)	7 (100)	7 (100)	0	5
Black Lava	4	0	NA	0	NA	NA	NA	NA	NA	NA	NA	NA	NA	NA
Chaos	4	4	4 (100)	8	Yes	4 (50.0)	4 (50.0)	4 (50.0)	8 (100)	4 (50.0)	4 (50.0)	8 (100)	4 (50.0)	3
Eternal Smoke [total]	4	4	4 (100)	8	Yes	8 (100)	6 (75.0)	8 (100)	8 (100)	8 (100)	8 (100)	8 (100)	6 (75.0)	7
Cool^g^	1	1	1 (100)	2	Yes	2 (100)	0	2 (100)	2 (100)	2 (100)	2 (100)	2 (100)	0	7
Extreme^g^	1	1	1 (100)	2	Yes	2 (100)	2 (100)	2 (100)	2 (100)	2 (100)	2 (100)	2 (100)	2 (100)	8^f^
Romance^g^	2	2	2 (100)	4	Yes	4 (100)	4 (100)	4 (100)	4 (100)	4 (100)	4 (100)	4 (100)	4 (100)	8^f^
Fantasia [total]	16	8	7 (43.8)	15	No	15 (100)	15 (100)	8 (53.3)	15 (100)	15 (100)	15 (100)	15 (100)	8 (53.3)	6
Castros Blend^g^	4	0	NA	NA	NA	NA	NA	NA	NA	NA	NA	NA	NA	NA
Formula Series^g^	4	0	NA	NA	NA	NA	NA	NA	NA	NA	NA	NA	NA	NA
Ice^g^	4	4	3 (75.0)	7	No	7 (100)	7 (100)	0	7 (100)	7 (100)	7 (100)	7 (100)	0	6
Regular^g^	4	4	4 (100)	8	No	8 (100)	8 (100)	8 (100)	8 (100)	8 (100)	8 (100)	8 (100)	8 (100)	7
Fumari	4	1	1 (25.0)	2	Yes	2 (100)	2 (100)	2 (100)	2 (100)	2 (100)	2 (100)	2 (100)	2 (100)	7
Gold Star	4	1	0	1	No	0	0	0	0	0	0	1 (100)	0	1
Haze	4	0	NA	0	NA	NA	NA	NA	NA	NA	NA	NA	NA	NA
HookaFina [total]	8	7	7 (87.5)	14	Yes	6 (42.9)	14 (100)	8 (57.1)	6 (42.9)	14 (100)	14 (100)	14 (100)	0	4
BLAK^g^	4	4	4 (100)	8	Yes	0	8 (100)	8 (100)	0	8 (100)	8 (100)	8 (100)	0	6
Gold^g^	4	3	3 (75.0)	7	Yes	6 (100)	6 (100)	0	6 (100)	6 (100)	6 (100)	6 (100)	0	6
Hooked on Hookah	4	4	0	4	Yes	0	0	4 (100)	4 (100)	4 (100)	4 (100)	4 (100)	0	5
Inhale	4	4	0	4	No	0	4 (100)	4 (100)	4 (100.0	0	4 (100)	4 (100)	0	5
Karizma	4	4	4 (100)	8	Yes	8 (100)	8 (100)	8 (100)	8 (100)	8 (100)	8 (100)	8 (100)	8 (100)	8^f^
Khalil Mamoon	4	0	NA	0	NA	NA	NA	NA	NA	NA	NA	NA	NA	NA
Lavoo	4	4	0	4	No	4 (100)	4 (100)	0	4 (100)	4 (100)	4 (100)	4 (100)	0	6
MYA	4	4	4 (100)	8	Yes	4 (50.0)	8 (100)	8 (100)	8 (100)	8 (100)	8 (100)	8 (100)	4 (50.0)	7
Malaki	4	4	4 (100)	8	Yes	0	8 (100)	8 (100)	8 (100)	8 (100)	8 (100)	8 (100)	0	7
Mazaya	4	4	4 (100)	8	No	8 (100)	8 (100)	8 (100)	8 (100)	4 (50.0)	8 (100)	8 (100)	4 (50.0)	7
Nakhla [total]	17	0	NA	0	NA	NA	NA	NA	NA	NA	NA	NA	NA	NA
El Basha^g^	4	0	NA	NA	NA	NA	NA	NA	NA	NA	NA	NA	NA	NA
Mix^g^	4	0	NA	NA	NA	NA	NA	NA	NA	NA	NA	NA	NA	NA
Mizo^g^	2	0	NA	NA	NA	NA	NA	NA	NA	NA	NA	NA	NA	NA
Regular^g^	4	0	NA	NA	NA	NA	NA	NA	NA	NA	NA	NA	NA	NA
Tropicana, Sheherazade^g^	3	0	NA	NA	NA	NA	NA	NA	NA	NA	NA	NA	NA	NA
Over Dozz	4	4	4 (100)	8	Yes	8 (100)	8 (100)	8 (100)	8 (100)	8 (100)	8 (100)	8 (100)	8 (100)	8^f^
Romman	4	0	NA	0	NA	NA	NA	NA	NA	NA	NA	NA	NA	NA
Rosetta	4	0	NA	0	NA	NA	NA	NA	NA	NA	NA	NA	NA	NA
Shisha Royale	4	0	NA	0	NA	NA	NA	NA	NA	NA	NA	NA	NA	NA
Social Smoke	4	1	1 (25.0)	2	No	2 (100)	2 (100)	2 (100)	2 (100)	2 (100)	2 (100)	2 (100)	2 (100)	7
Starbuzz [total]	12	9	5 (41.7)	14	No	6 (42.9)	13 (92.9)	14 (100)	14 (100)	10 (71.4)	14 (100)	14 (100)	6 (42.9)	4
Exotic^g^	4	3	3 (75.0)	6	Yes	6 (100)	6 (100)	6 (100)	6 (100)	6 (100)	6 (100)	6 (100)	6 (100)	7
Serpent^g^	4	4	0	8	No	0	4 (100)	4 (100)	4 (100)	0	4 (100)	4 (100)	0	5
Vintage^g^	4	2	2 (50.0)	4	No	0	3 (75.0)	4 (100)	4 (100)	4 (100)	4 (100)	4 (100)	0	5
Tangiers	4	3	3 (75.0)	6	Yes	6 (100)	6 (100)	6 (100)	6 (100)	6 (100)	6 (100)	6 (100)	6 (100)	7
TickTock	4	0	NA	0	NA	NA	NA	NA	NA	NA	NA	NA	NA	NA
Ugly Hookah	4	0	NA	0	NA	NA	NA	NA	NA	NA	NA	NA	NA	NA
Zumerret	4	1	1 (25.0)	2	Yes	2 (100)	0	2 (100)	2 (100)	2 (100)	2 (100)	2 (100)	0	6
Total/Mean^h^	181	97	68 (52.1)	165	56.5	120 (68.9)	147 (79.0)	132 (76.5)	147 (85.7)	144 (81.4)	160 (93.5)	165 (100)	84 (44.4)	5.7

Abbreviation: NA, not applicable.

^a^ Denominator for 2 nicotine warnings per package was equal to the number of packages.

^b^ Denominator for warnings in correct location equal to the number of brands with at least 1 nicotine warning on a package.

^c^ Denominator for 30% panel through formatting compliant was equal to the number of packages with nicotine warnings plus the number of packages with 2 nicotine warnings per package.

^d^ Number of elements for 100% formatting compliance was 7 (30% panel through formatting compliant).

^e^ Number of elements for Cumulative Compliance Score was 8 (7 formatting elements plus 2 nicotine warnings per package in the correct location). Score range: 0 to 8, with higher scores indicating more compliant components.

^f^ Brands with 100% formatting compliance. All packages have 2 US Food and Drug Administration nicotine warnings.

^g^ Individual product lines are unique product lines within the umbrella brand. If there are 2 names in the product line cell, the packaging was the same and, thus, the products are considered as a single product line for this study.

^h^ Total/mean was calculated by brand and excludes individual product lines.

## Discussion

As of 2020, 2 years after the FDA mandated warnings on all WT packages sold in the US, compliance with this federal regulation was low. To our knowledge, this study was the first to assess compliance with warning requirements on WT packaging in the US. Study limitations are that only 33 brands out of the 66 identified were assessed and that packages were purchased in 2020.

The FDA should consider issuing warning letters or enforcement actions to WT manufacturers to increase policy compliance. Future studies should assess the implications of WT warnings for consumer perceptions and behaviors.

## Notes

Supplement.
Data Sharing Statement
