# Supplementary material for: Compliance With US Federal Regulations on Waterpipe Tobacco Warnings on Packaging
Source: JAMA Netw Open. 2024 Feb 2;7(2):e2354467. doi: 10.1001/jamanetworkopen.2023.54467 (PMC10837745; doi:10.1001/jamanetworkopen.2023.54467)
Supplement: Supplement. — Data Sharing Statement [file jamanetwopen-e2354467-s001.pdf]

Data Sharing Statement

Ross. Compliance With US Federal Regulations on Waterpipe Tobacco Warnings on Packaging. *JAMA Netw Open*. Published February 02, 2024. doi:10.1001/jamanetworkopen.2023.54467

Data
Data available: No

Additional Information
Explanation for why data not available: We will make the data upon reasonable request to the study PI, Dr. Sutfin.
